# Brain and Optic Chiasm Herniation into Sella after Pituitary Tumor Apoplexy

**DOI:** 10.3389/fendo.2017.00192

**Published:** 2017-08-07

**Authors:** Maria M. Pineyro, Patricia Furtenbach, Ramiro Lima, Saul Wajskopf, Nicolas Sgarbi, Raul Pisabarro

**Affiliations:** ^1^Clínica de Endocrinología y Metabolismo, Hospital de Clínicas, Facultad de Medicina, Universidad de la República, Montevideo, Uruguay; ^2^Neurocirugía, Hospital de Clínicas, Facultad de Medicina, Universidad de la República, Montevideo, Uruguay; ^3^Imagenología, Hospital de Clínicas, Facultad de Medicina, Universidad de la República, Montevideo, Uruguay

**Keywords:** brain herniation, optic chiasm herniation, pituitary apoplexy, prolactinoma, empty sella

## Abstract

Brain and optic chiasm herniation has been rarely reported following dopamine agonist treatment for large prolactinomas. We report a case of brain and optical chiasm herniation, secondary to an empty sella due to apoplexy of a prolactinoma, and we focus on the specific presentation of this case. A 32-year-old female presented to a neurologist complaining of headaches. Her past medical history was significant for acute vision loss in both eyes accompanied by right third nerve palsy when she was 16 years old. She does not recall any endocrine or imaging evaluation at that time and she had spontaneous partial recovery of left eye vision within 3 months, with permanent blindness of right eye. She did not return to any follow-up until her neurologist consultation. Brain magnetic resonance imaging (MRI) revealed herniation of frontal lobe and optic chiasm into the pituitary sella, as well as a pituitary hypointense lesion measuring 5 mm × 5 mm after gadolinium injection. Prolactin levels were 206 ng/ml (4.79–23.3 ng/ml). Repeated prolactin was 258 ng/ml (4.79–23.3 ng/ml). She was started on bromocriptine 2.5 mg/day. Prolactin levels and menstrual cycles normalized. A repeat brain MRI performed 5 months later showed disappearance of pituitary mass, with no changes in brain and chiasmal herniation. To our knowledge, this is the first reported case of brain associated with chiasm herniation secondary to pituitary apoplexy of a prolactinoma. In conclusion, this case highlights that frontal lobe herniation in combination with optic chiasm herniation can be a complication of pituitary tumor apoplexy. Long-term surveillance of patients with pituitary apoplexy is warranted to detect delayed complications.

## Introduction

Prolactinomas are the most common pituitary adenomas, accounting for approximately 50% of all pituitary tumors (1). Dopamine agonists are the gold standard treatment controlling hormone secretion and tumor growth in most cases. Herniation of the suprasellar visual system into a secondary empty sella created by tumor involution has been reported as a side effect of dopamine agonists’ treatment (2, 3).

Simultaneous brain and optical chiasm herniation has been rarely reported following dopamine agonist treatment for large prolactinomas (4–7). Empty sella as well as herniation of the suprasellar visual system due to pituitary apoplexy has been seldom reported (8–10). We report a case of brain and optical chiasmal herniation, secondary to an empty sella due to apoplexy of a prolactinoma.

## Background

A 32-year-old female presented to a neurologist complaining of a long history of holocranial oppressive headaches, of severe intensity, with a frequency of 2 or 3 per week, without any accompanying symptoms, which resolved initially with NSAIDs. Her past medical history was significant for acute vision loss of both eyes accompanied by right third nerve palsy when she was 16 years old. She remembers headache at that time, but not its characteristics and did not describe it as severe or the “worst headache of her life.” In addition, she does not recall any endocrine or imaging evaluation at that time. She refers spontaneous partial recovery of left eye vision within 3 months, with permanent blindness of right eye. She did not return to follow-up visits until her neurologist consultation. Thelarche started at age 11–12 years, and menarche started at the age of 30 years, followed by oligomenorrhea. She denied galactorrhea or other endocrine symptoms. She did not present olfactory problems.

**Figure 1:**
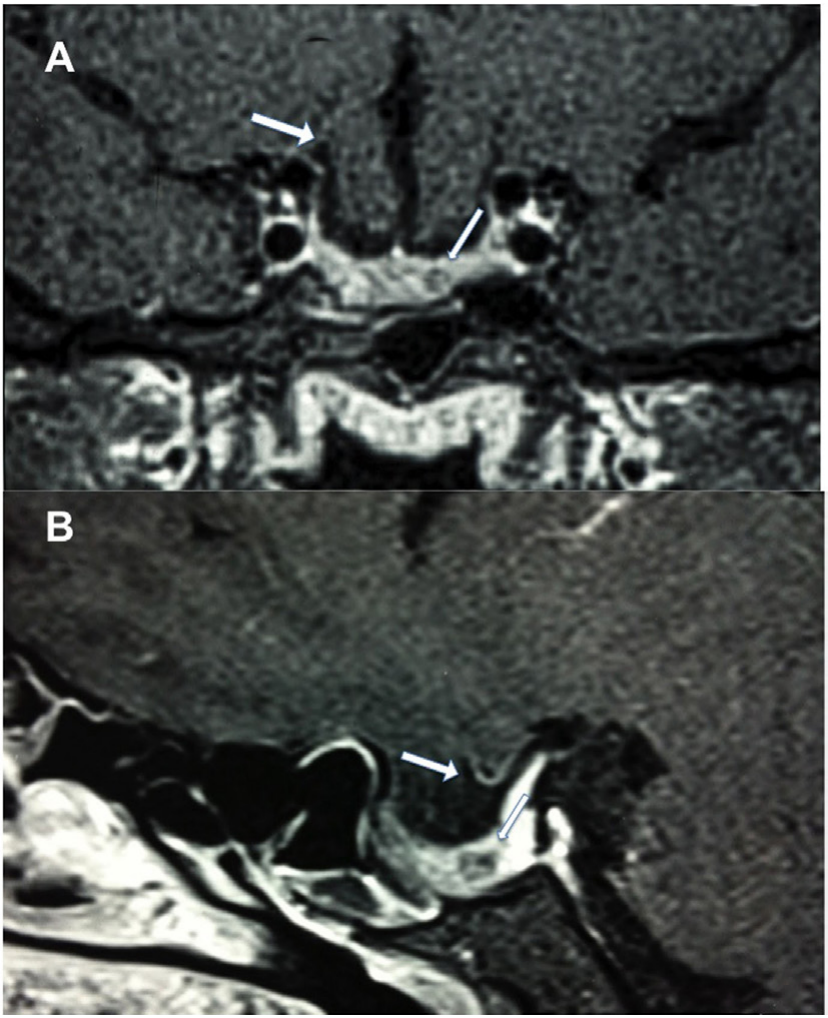
Postcontrast T1-weighted magnetic resonance imaging. **(A)** Coronal view showing frontal lobe herniation (thick arrow) and a 5 mm hypointense lesion (thin arrow), and **(B)** sagittal view showing chiasm herniation and a 5 mm hypointense lesion.

**Figure 2:**
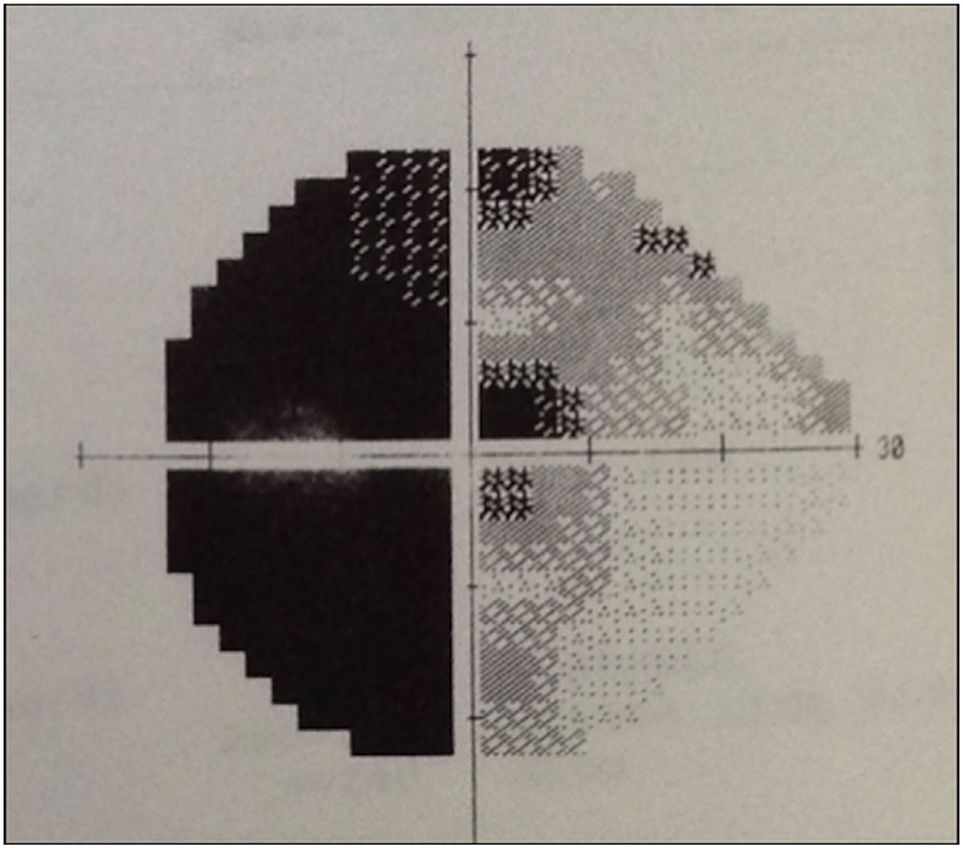
Automated perimetry showing left temporal hemianopia.

**Figure 3:**
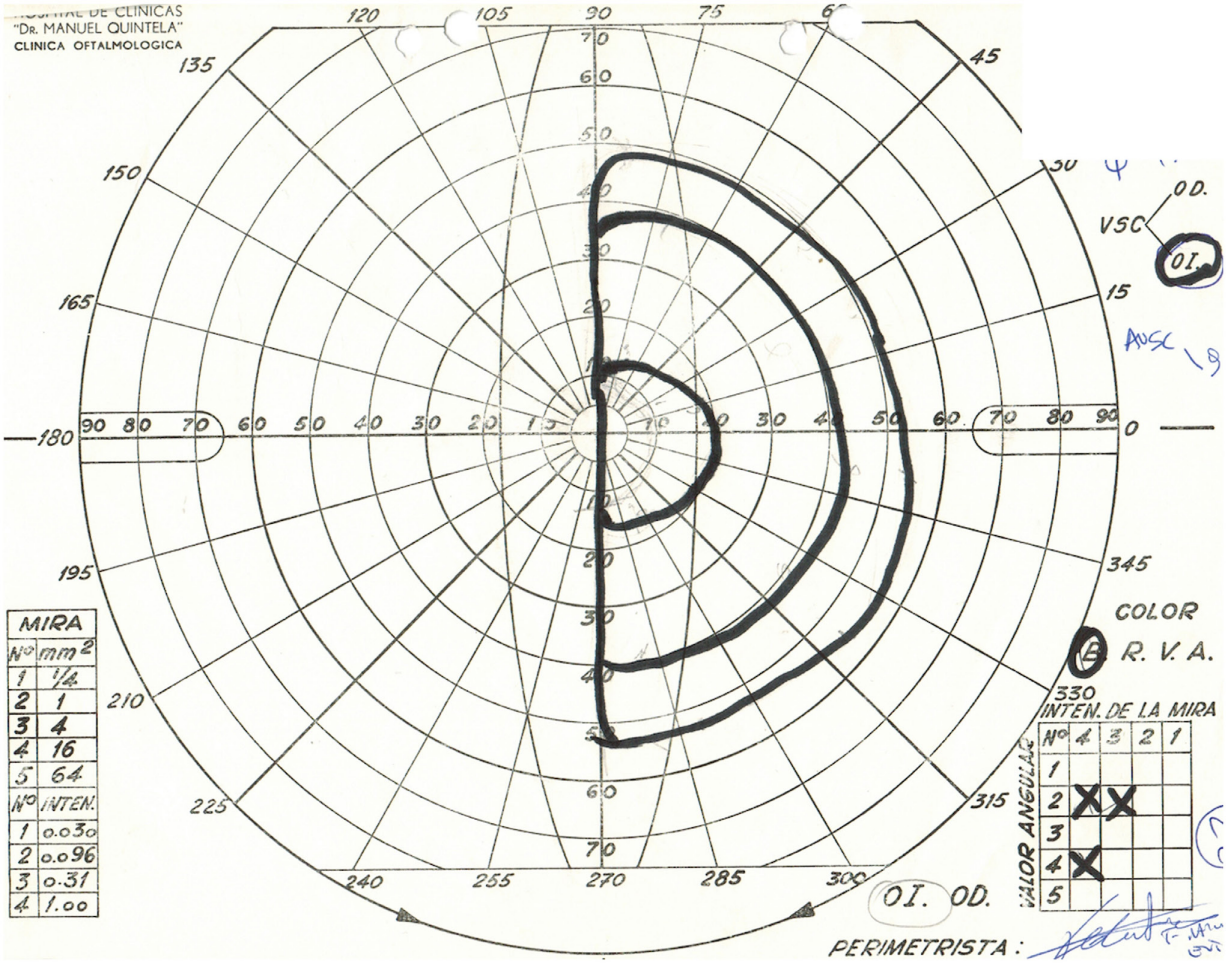
Goldmann perimetry showing left temporal hemianopia.

Physical examination revealed obesity [weight was 79 kg (174 lbs) and height 1.60 m], with a BMI of 30.8. Neurology examination revealed blindness of the right eye, left temporal hemianopia, and complete right third nerve palsy. Brain MRI showed herniation of frontal lobe and optic chiasm into the pituitary sella, as well as a pituitary hypointense lesion measuring 5 mm × 5 mm after gadolinium injection (Figure 1) and she was referred to our department. Laboratory tests showed elevated prolactin level of 206 ng/ml, repeated 258 ng/ml (*N* 4.79–23.3 ng/ml). In addition, secondary hypothyroidism was diagnosed with low FT4 of 0.70 ng/dl (*N* 0.93–1.71) and inappropriately normal TSH of 3.61 μUI/ml (*N* 0.27–4.20). ACTH stimulation test was normal. At diagnosis, FSH was 5.3 mUI/ml (3.5–12.5 mUI/ml), LH was 3.1 mUI/ml (*N* 2.4–12.6 mUI/ml), and estradiol 22 pg/ml (12.5–165.5 pg/ml). Age-adjusted IGF-1 was normal. Formal visual field test showed left bitemporal hemianopia (right eye blindness) (Figures 2 and 3).

**Figure 4:**
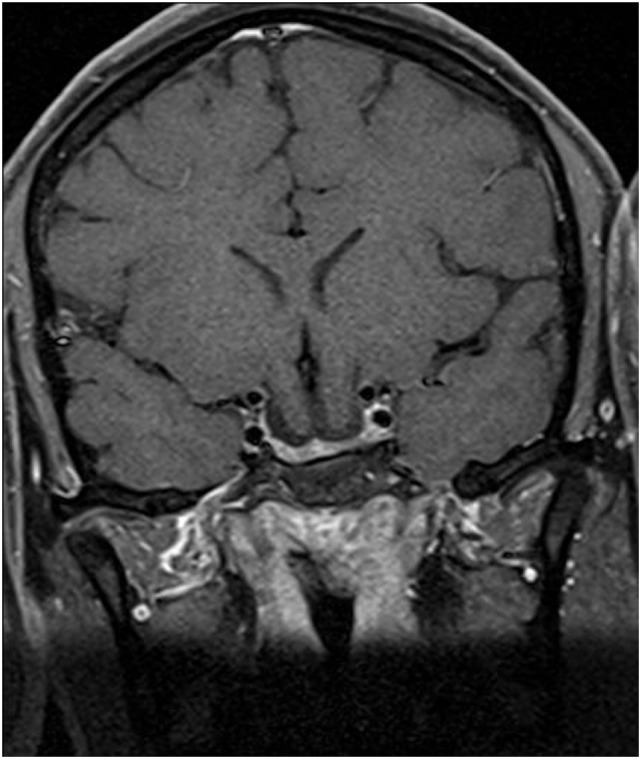
Coronal postcontrast T1-weighted image 5 months after therapy with bromocriptine.

She was started on bromocriptine 2.5 mg/day and levothyroxine 50 mcg/day. After 1 month of treatment menstrual cycles normalized. Vision remained unchanged. Two months after therapy, prolactin level was 18.1 ng/ml (N 4.79–23.3) and FT4 levels were within normal range. A repeat brain MRI performed 5 months later showed disappearance of pituitary lesion, with no changes in brain and chiasmal herniation (Figure 4). At this point she is concerned about fertility and potential pregnancy risks.

## Discussion

We report a rare case of brain and optic chiasmal herniation secondary to empty sella due to pituitary apoplexy of a prolactinoma. Pituitary apoplexy refers to a rare clinical syndrome that occurs as a consequence of infarction and/or hemorrhage of the pituitary gland, most often involving a pituitary adenoma. Reported prevalence is approximately 6.2 cases per 100,000 inhabitants (1), with 2–12% of pituitary tumors experiencing this complication (11–15). Prevalence appears to be lower in prolactinomas (16). Asymptomatic apoplexy is much more common, with up to 25% of pituitary tumors showing areas of hemorrhage and/or necrosis (13, 17, 18).

The clinical manifestations of pituitary apoplexy are variable, depending on extension of hemorrhage or infarction. The most frequent symptom is headache, present in up to 100% of patients. Although it is commonly described as being sudden and severe, it may also be subacute (15). Headache is believed to result from dural traction or due to passage of blood into the subarachnoid space causing meningeal irritation. Compression of optic chiasm or optic nerves by the sellar mass causes visual impairment in up to 75% of patients, most often bitemporal hemianopia. Blindness can happen, although rare (19). Oculomotor palsies occur in approximately half of the patients, with the third cranial nerve being the most frequently affected (20, 21). Retrospectively, though not formal diagnosis, this patient had classic symptoms of pituitary apoplexy such as headache, impaired vision, and ophthalmoplegia.

In addition, hypopituitarism is a major manifestation of pituitary apoplexy, with up to 80% of cases presenting with deficit of one or more anterior pituitary hormones (22). This is probably due to pituitary and stalk compression, destruction of anterior pituitary, or pre-existing deficiencies. ACTH deficiency is the most common deficit, detected in approximately 50–80% of cases (23, 24). Gonadotropin and thyrotropic deficiency are found in 76–79% and 50–57%, respectively (22). In this case, we do not know endocrine status at the time of presentation. However, she had delayed puberty probably secondary to gonadotropin suppression due to hyperprolactinemia, as her menstrual cycles were restored when prolactin levels normalized. She either did not suffer from ACTH deficiency or this axis later recovered. Improvement in pituitary function has been reported weeks and months after the event (25), even in patients who received conservative treatment (21). On the other hand, pituitary axes should be monitored as hypopituitarism may develop later in time (25), as may have happened in this case with secondary hypothyroidism.

Empty sella and suprasellar visual system herniation have been rarely reported following pituitary apoplexy. Yücesoy et al. (9) reported a patient who presented with severe headache, nausea, vomiting, and visual loss. Brain MRI and cranial computerized tomography scan showed pituitary apoplexy and subarachnoid hemorrhage. Four weeks later visual loss continued to progress. Repeated MRI demonstrated an empty sella. In addition, L’Huillier et al. (10) reported one case of empty sella in seven pituitary adenomas following pituitary apoplexy. Moreover, empty sella can be a consequence of silent hemorrhage or infarction of a pituitary tumor. Yoshino et al. (26) and Weiss (27) reported empty sella after spontaneous resolution of non-functioning pituitary adenomas.

Kaufman et al. (8) reported suprasellar visual system herniation in eight patients with secondary empty sella, and in one of these suspected etiology was a spontaneous apoplexy of a microadenoma.

We found only four reported cases of brain associated with optic chiasm herniation in large prolactinomas following dopamine agonist treatment (4–7).

Bangash et al. (4) reported a 71-year-old man with a macroprolactinoma treated with bromocriptine increased to 5 mg TID. In an annual follow-up imaging right frontal lobe herniation with optic chiasmal deformation was seen, with no residual tumor. His medical treatment was unchanged, with stable vision.

Dhanwal and Sharma (5) reported a 32-year-old man with a giant prolactinoma treated with cabergoline beginning with 1 mg/week with gradual increase, receiving 3 mg per week at 3 months and thereafter. Six months after he presented two episodes of generalized seizures and formal visual field test deterioration. Magnetic resonance imaging (MRI) disclosed herniation of the frontal lobe and optic chiasm into the pituitary fossa. He was managed with reduction of cabergoline dose to 2 mg per week, with marginal improvement in his vision.

Zhang et al. (7) reported a 24-year-old woman with a macroprolactinoma treated with bromocriptine, increased to 2.5 mg TID. Two years later she presented with severe visual field deterioration. MRI showed inferior frontal lobe and optic chiasm herniation. She underwent transsphenoidal endoscopic packing or chiasmopexy with improvement in visual field defects.

Papanastasiou et al. (6) reported a 42-year-old man diagnosed with a giant prolactinoma treated with cabergoline starting with 1.5 mg/week and increasing 1 mg weekly till a total dose of 3.5 mg per week. Three months after treatment he presented with visual field deterioration. MRI revealed herniation of the right frontal lobe and optic chiasm into an empty sella. He was managed by cabergoline dose reduction to 1 mg per week. In addition, a craniotomy was done with excision of the adenoma and untethering of optic apparatus through a transglabelar approach. There was an improvement in visual field defect.

Recently, a case of pons herniation into the clivus after treatment of a giant prolactinoma with cabergoline has been reported. Moles Herbera et al. (28) reported a 59-year-old with a giant prolactinoma treated with cabergoline 1 mg per week. Eighteen months after treatment he presented with left hemiparesis and dysarthria. MRI revealed an anterior herniation of the pons and secondary enlargement of the fourth ventricle. His cabergoline dose was reduced. There was improvement in left lower limb strength.

Mechanism of brain herniation into an empty sella is unclear (4–6). The expansion of a macroadenoma may increase the opening through the diaphragma sella. Moreover, the expanding tumor may cause bone and meningeal erosion that can facilitate brain herniation when there is significant shrinkage of the tumor. In the reported cases (4–7), there was significant decrease in tumor size due to medical treatment of a prolactinoma. In our case, pituitary apoplexy may have contributed to increase the opening through the diaphragma sella, as it produces an increase in intrasellar pressure. In addition, pituitary apoplexy caused a subsequent decrease in tumor size by tumor infarction allowing the neural structures to herniate.

In addition, this patient could have a germline mutation in the aryl hydrocarbon receptor interacting protein (AIP) gene. This gene has been found to cause a hereditary predisposition to pituitary tumors and denotes an important cause of pituitary tumors in young patients (29). We did not screen for the AIP gene mutation, as this test is not available in our country.

We believe this patient does not have a hypothalamic malformation. Among them we review encephalocele as a possible cause of brain herniation.

Encephalocele refers to rare developmental abnormalities characterized by herniation of the brain and/or meninges through a skull defect. They can be classified as anterior (frontal, sincipital, and basal) and posterior. Basal encephalocele is the herniation of brain tissue through bony defects along the cribriform plate and body of sphenoid or ethmoid. It is the less frequent type (1.5%) of encephalocele. This uncommon congenital malformation has an estimated frequency of 1 in 35,000 to 40,000 live births (30). Basal encephalocele can be classified as transethmoidal and transsphenoidal types. The transsphenoidal type accounts for only 5% of all basal encephalocele (31). The third ventricle, including hypothalamus and optic chiasm, herniates through the sphenoid bone. This type can be either intrasphenoidal with extension into the sphenoid sinus but confined by the sinus floor or true transsphenoidal protruding through the floor of the sphenoid sinus into the nasopharynx or nasal cavity. Mechanisms proposed include defects of ossification, as well as persistence of craniopharyngeal canal (31, 32). It is often associated with midline craniofacial defects such as nasal cleft, cleft lip, cleft palate, hypertelorism, and optic and retinal malformations. The vast majority is diagnosed at birth or first year of life. However, there have been some cases diagnosed during adulthood, as facial abnormalities can be subtle or absent (33). In these cases, clinical presentation includes CSF rhinorrhea, visual impairment including visual field defects and amaurosis, endocrine abnormalities (hypopituitarism, hyperprolactinemia), and an epipharyngeal soft tissue mass (31, 34, 35).

We do not believe this patient has a basal encephalocele. First, although this patient has a brain and chiasm herniation into the sella, it is confined by the sphenoid bone and does not go through into the sphenoid sinus. Second, she does not have any midline craniofacial defects. Third, she presented with classic symptoms of pituitary apoplexy, some of which have not been reported associated with basal encephalocele (third nerve palsy). In addition, the acuteness of clinical presentation is suggestive of pituitary apoplexy, with symptoms of basal encephalocele being most often insidious.

In addition, we believe this patient does not have other hypothalamic malformation. She does not present clinical features, laboratory findings or imaging features consistent with brain or pituitary and parasellar region congenital malformations (36–38).

To our knowledge, this is the first reported case of brain associated with chiasm herniation secondary to pituitary apoplexy of a prolactinoma. Pregnancy effects on brain herniation are unknown.

## Concluding Remarks

This case highlights that frontal lobe herniation in combination with optic chiasm herniation can be a complication of pituitary tumor apoplexy. Long-term follow-up of patients with pituitary apoplexy is needed to determine possible late-onset complications.

## Ethics Statement

The patient provided written informed consent for research participation as well as for the publication of indirectly identifiable data (age, gender, and medical history).

## Author Contributions

Wrote the first draft of the manuscript: MP. Contributed to the writing of the manuscript: PF. Made contributions to the acquisition of the clinical data: MP, PF, RL SW, NS, and RP. Agreed with manuscript results and conclusions: MP, PF, RL, SW, NS, and RP. Made critical revisions and approved final version: MP. All the authors revised and approved the final manuscript and agreed to be accountable for the content of the work.

## Conflict of Interest Statement

The authors declare that the research was conducted in the absence of any commercial or financial relationships that could be construed as a potential conflict of interest.
